# Exosomal microRNA-144 from bone marrow-derived mesenchymal stem cells inhibits the progression of non-small cell lung cancer by targeting CCNE1 and CCNE2

**DOI:** 10.1186/s13287-020-1580-7

**Published:** 2020-02-26

**Authors:** Yuan Liang, Dalin Zhang, Linlin Li, Tian Xin, Yuwei Zhao, Rui Ma, Jiang Du

**Affiliations:** 1 Medical Oncology Department of Thoracic Cancer (2), Cancer Hospital of China Medical University, Liaoning Cancer Hospital & Institute, No. 44, Xiaoheyan Road, Dadong District, Shenyang, 110042 Liaoning Province People’s Republic of China; 2 Department of Thyroid Surgery, The First Affiliated Hospital of China Medical University, Shenyang, 110001 People’s Republic of China; 3 Department of Pathology, The First Affiliated Hospital and College of Basic Medical Science, China Medical University, No. 155, Nanjing North Street, Heping District, Shenyang, 110001 Liaoning Province People’s Republic of China

**Keywords:** Non-small cell lung cancer, Bone marrow-derived mesenchymal stem cells, Exosomes, MicroRNA-144, Cyclin E1, Cyclin E2

## Abstract

**Background:**

Mesenchymal stem cells (MSCs) are pluripotent mesenchymal cells present in various adult tissues. MSCs secrete exosomes as regulators of the tumor niche, with involvement in tumorigenesis and metastasis. The regulatory role of microRNAs (miRs or miRNAs) in MSCs via targeting cyclin E1 (CCNE1) or cyclin E2 (CCNE2) has been extensively reported. Since exosomes are considered as protective and enriched sources of shuttle miRNAs, we hypothesized that exosomal transfer of miR-144 from bone marrow-derived MSCs (BMMSCs) would affect the development of non-small cell lung cancer (NSCLC) cells by targeting CCNE1 and CCNE2.

**Methods:**

We first quantified the levels of miR-144, CCNE1, and CCNE2 in NSCLC tissues and cell lines and then undertook gain- and loss-of-function studies of miR-144, CCNE1, and CCNE2 to investigate their roles in the biological characteristics of NSCLC in vitro. NSCLC cells (A549) were exposed to exosomes derived from MSCs, and cell proliferation and colony formation rate were determined using in vitro assays. Finally, effects of BMMSC-derived exosomal miR-144 on tumor development were studied in vivo.

**Results:**

In NSCLC tissues and cell lines, miR-144 was expressed poorly and CCNE1 and CCNE2 were expressed highly. Artificially elevating miR-144 inhibited cell proliferation, colony formation, and the number of S phase-arrested cells in NSCLC by downregulating CCNE1 and CCNE2. Additionally, BMMSC-derived exosomal miR-144 led to restrained NSCLC cell proliferation and colony formation. These inhibitory effects of BMMSC-derived exosomes carrying miR-144 on NSCLC were confirmed by experiments in vivo.

**Conclusion:**

Collectively, these findings revealed inhibitory effects of BMMSC-derived exosomal miR-144 on NSCLC progression, which were mediated by downregulation of CCNE1 and CCNE2.

## Background

Statistical analysis from the American Cancer Society and the Cancer Statistics Center shows that each year more than 150,000 patients die from lung cancer, and 200,000 new cases are diagnosed [1]. Primary lung cancer is traditionally classified into small cell lung cancer and non-small cell lung cancer (NSCLC) [2]. Surgical resection is the main treatment method for lung cancer, but most patients are diagnosed at the advanced disease stage, when relatively ineffective drug therapy is the only feasible treatment [2]. Mesenchymal stem cells (MSCs) are non-hematopoietic progenitor cells that can be obtained from bone marrow aspirates or adipose tissue, and then expanded and genetically modified in vitro, presenting a potential therapeutic strategy for cancer patients [3]. The molecular mechanisms behind the function of MSCs in tumorigenesis and cancer development are not well-defined, but better knowledge of these mechanisms could facilitated improved prognosis and suppressed malignancy.

Cyclin E1 (CCNE1) is an important factor that can regulate the entry of proliferating cells into S and G1 phases, which is reported to play a role in regulating growth of lung cancer cells [4]. A previous study revealed that the cancer cell cycle progression was inhibited by ZYG-11 family member A (ZYG11A), which targets CCNE1 [5]. Meanwhile, downregulation of cyclin E2 (CCNE2) is demonstrated to notably restrain NSCLC cell function [6]. Overexpression of CCNE2 antagonizes the inhibitory effect of microRNA (miR)-30d-5p on the proliferation and motility abilities of NSCLC cells [6]. Collectively, the miRNAs are a class of small non-coding RNA molecules consisting of 20–23 nucleotides, which plays crucial roles in many biological processes by binding to certain target mRNAs, thus restraining their translation or degradation [7]. Numerous reports have indicated that miRNAs are dysregulated in a variety of tumors and might therefore be used as plasma diagnostic markers for cancers [8]. It was previously reported that inhibition of miR-144 may enhance the metastasis of NSCLC [9].

Exosomes are small lipid bilayer-enclosed particles with a diameter of 30–140 nm, which originate in the endosomes [10] and are released from various cell types under specific physiological or pathological conditions. Exosomes can transport multiple functional molecules, including mRNAs and miRNAs [11]. They have been identified as mediators of interactions among cancer cells and can function as long range signal effectors potentially coordinating tumor formation, progression, metastasis, drug resistance, tube formation, and tumor immunity [12]. We now test the hypothesis that BMMSC-derived exosomes carrying miR-144 can affect NSCLC growth and progression by targeting CCNE1 and CCNE2.

## Methods

### Ethics statement

This experiment was approved by the Ethics Committee of Cancer Hospital of China Medical University. All participants enrolled in the study signed informed written consent documents, and the experiment procedures were in line with the *Declaration of Helsinki*. All animal experiments were in strict accordance with the recommendations in the Guide for the Care and Use of Laboratory Animals of the National Institutes of Health.

### Microarray-based gene expression profiling

Gene expression profiling (GSE74706, GSE33532) and miRNA profiling (GSE102286) associated to NSCLC were retrieved from the Gene Expression Omnibus database. We used the limma package of R language to screen the differentially expressed genes (DEGs) and miRNAs and the DAVID package for functional enrichment analysis of DEGs. The ggplot2 package in R language was utilized for correlational analysis of gene expression correlation and for visualization. The potential regulatory relationship between DEGs and miRNAs was predicted using the microRNA.org, miRDB, TargetScan, mirDIP, and DIANA databases. We calculated Venn diagrams to identify the overlap between different genes and miRNA data in the database.

### Patient enrollment

Sixty-eight patients (46 males and 22 females, 57.28 ± 4.58 years old) diagnosed with NSCLC at the Cancer Hospital of China Medical University were enrolled in this study. None of the patients had received local or systemic chemoradiotherapy before their surgery. Resected tumor tissues and adjacent normal tissues (5 cm away from tumor tissues) were collected from each patient and immediately stored in liquid nitrogen. Bone marrow specimens were also collected from three inpatients (2 males and 1 female) with osteonecrosis of the femoral head, aged from 26 to 52 years, without loss of height of the femoral head or diseases such as trauma, cardiovascular disease, or tumor invasion according to magnetic resonance imaging examinations.

### Immunohistochemistry (IHC)

The paraffin-embedded tissues were sliced, dewaxed, dehydrated with gradient ethanol, and immersed in 3% H_2_O_2_. Next, the sections were subjected to antigen retrieval and then blocked with normal goat serum (C-0005, Shanghai Haoran Bio Technologies Co., Ltd., Shanghai, China) for 20 min. The sections were subsequently incubated with primary antibody mouse anti-CCNE1 (1:100, ab238081), CCNE2 (1:50, ab32103), and KI-67 (1:150, ab156956) at 4 °C overnight. The sections were further incubated with secondary antibody goat anti-mouse immunoglobulin G (IgG) (1:1000, ab6785, Abcam Inc., Cambridge, MA, UK) at 37 °C for 20 min, followed by incubation with peroxidase horseradish-labeled streptavidin ovalbumin solution at 37 °C for 20 min. After being developed with diaminobenzidine, the sections were counterstained by hematoxylin (PT001, Shanghai Bogoo Biotechnology., Co., Ltd., Shanghai, China) for 1 min, blued with ammonia water, and then dehydrated with gradient ethanol, cleared by xylene, sealed by neutral balsam, and observed under a microscope.

### Cell treatment

NSCLC cell lines A549, NCI-H1975, NCI-H1299, and SPC-A1 and normal human bronchial epithelial (HBE) cell were purchased from Bena Culture Collection (Suzhou, China). The cells were cultured in Dulbecco’s modified Eagle’s medium (DMEM; 12800017, Gibco, Carlsbad, CA, USA) containing 10% fetal bovine serum (FBS, 26140079, Gibco, Carlsbad, CA, USA) at 37 °C with 5% CO_2_ and saturated humidity. The cells (1 × 10^5^ cells/mL) were seeded into 6-well plates and cultured for 24 h. Cells were then transfected with plasmids of miR-144 mimic, miR-144 inhibitor, short hairpin (sh)-CCNE1, sh-CCNE2, or their relative negative control (NC) individually or together, using Lipofectamine 2000 reagent (Invitrogen Inc., Carlsbad, CA, USA) according to the manufacturer’s instructions. The plasmids of sh-NC, sh-CCNE1, and sh-CCNE2 were purchased from Guangzhou RiboBio Co., Ltd. (Guangzhou, Guangdong China), and plasmids of mimic-NC, miR-144 mimic, inhibitor-NC, and miR-144 inhibitor were from Shanghai GenePharma Co., Ltd. (Shanghai, China). BMMSCs and NSCLC cells were transfected by Lipofectamine 2000. Then, a 250-μL volume of Opti-minimum essential medium (MEM) (Gibco, Grand Island, NY, USA) was used to dilute 4 μg target plasmids and 10 μL Lipofectamine 2000 respectively. After mixing by gentle shaking, the samples were allowed to stand for 5 min at room temperature. Next, the two liquids were mixed for 20 min of reaction and then added to the cell culture wells. The culture plate was shaken, and then placed in a 5% CO_2_ incubator at 37 °C for further culture. After 6 h, the culture medium was changed, and cells were collected after 36–48 h of transfection for subsequent experiments.

### Reverse transcription quantitative polymerase chain reaction (RT-qPCR)

The total RNA was extracted using a Trizol Kit (15596026, Invitrogen, Carlsbad, CA, USA) and reversely transcribed into complementary DNA by PrimeScript RT reagent Kit (RR047A, Takara Bio Inc., Otsu, Japan). RT-qPCR was then performed with Fast SYBR Green PCR kit (Applied Biosystems, Carlsbad, CA, USA) on an ABI 7500 instrument (Applied Biosystems, Carlsbad, CA, USA). The relative expression of genes was calculated by means of relative quantification (2^−△△Ct^ method) with glyceraldehyde-3-phosphate dehydrogenase (GAPDH) and U6 as internal controls. The primer sequences are shown in Table 1.

**Table 1:** Primer sequences for RT-qPCR

Gene	Primer sequence (5′-3′)
miR-144	F: 5′-TCCGATCATGTAGTAGATATTGACAT-3′
	R: 5′-GTGCAGGGTCCGAGGT-3′
CCNE1	F: 5′-GTGGCTCCGACCTTTCAGTC-3′
	R: 5′-CACAGTCTTGTCAATCTTGGCA-3′
CCNE2	F: 5′-GCATTATGACACCACCGAAGA-3′
	R: 5′-TAGGGCAATCAATCAATCACAGC-3′
U6	F: 5′-TCTTTGGAATTCAAGGTCGGGCAGGAAGAGGGCCTA-3′
	R: 5′-CGCGGATCCTAGTATATGTGCTGCCGAAGC-3′
GAPDH	F: 5′-GAAGGTGAAGGTCGGAGT-3′
	R: 5′-GAAGATGGTGATGGGATTTC-3′

RT-qPCR, reverse transcription quantitative polymerase chain reaction; *F* forward, *R* reverse, *miR-144* microRNA-144, *CCNE1* cyclin E1, *CCNE2* cyclin E2, *GAPDH* glyceraldehyde-3-phosphate dehydrogenase

### Western blot analysis

The total protein content was isolated with an enhanced radio immunoprecipitation assay lysis buffer (Wuhan Boster Biological Technology Co., Ltd., Wuhan, China). The proteins were separated by sodium dodecyl sulfate-polyacrylamide gel electrophoresis and then transferred to a polyvinylidene fluoride membrane. After being blocked in sealing solution, the membrane was incubated with the primary antibodies rabbit anti-human CCNE1 (1:2000, ab33911), CCNE2 (1:500, ab32103), KI67 (1: 000, ab92742), proliferating cell nuclear antigen (PCNA) (1:1000, ab925522), or GAPDH (1:5000, ab181602, all from Abcam Inc., Cambridge, MA, USA), which served as a NC, at 4 °C overnight. The next day, the membrane was incubated with secondary goat anti-rabbit IgG (1:10000, ab205718, Abcam Inc., Cambridge, MA, USA) at 37 °C for 1 h. The samples were developed using ECL reaction solution, photographed using SmartView Pro 2000 (UVCI-2100, Major Science, Saratoga, CA, USA), followed by gray scale analysis of the protein band pattern using the Quantity One software.

### Dual luciferase reporter assay

The 3′ untranslated regions (UTRs) of CCNE1 and CCNE2, which contain potential miR-144 binding sites, were constructed into the PGLO vector (PGLO-CCNE1 wild type (WT) and PGLO-CCNE2 WT). The mutant (MUT) forms, in which the potential miR-144 binding sites were mutated for loss of function, were also constructed (PGLO-CCNE1 MUT and PGLO-CCNE2 MUT). Report plasmids were co-transfected with miR-144 mimic, or miR-NC into HEK293T cells. After 24 h of transfection, the cells were lysed and centrifuged, and the supernatant was collected. The luciferase activity was detected using Dual-Luciferase® Reporter Assay System (E1910, Promega Corp., Madison, WI, USA) according to the manufacturer’s instructions.

### Isolation and identification of BMMSCs

BMMSCs were isolated from the three bone marrow donations as previously reported [13] and cultured in DMEM-F12 (Hyclone, South Logan, UT, USA) containing 10% FBS (10099141, Gibco, Carlsbad, CA, USA) and 0.2% penicillin and streptomycin (Hyclone, South Logan, UT, USA). Then, the cells were passaged every 3 days, and BMMSCs of the third to seventh passages were used for further experiments. The BMMSCs were cultured in BMMSCs’ osteogenic, adipogenic, and cartilage-differentiated OriCell™ medium (Cyagen Biosciences Inc., Guangzhou, China). Finally, the BMMSCs were stained with alizarin red and oil red O.

BMMSCs at the third passage were incubated with mouse monoclonal antibodies against CD105 (ab11414, 1:100), CD73 (ab81720, 1:50), CD90 (ab23894, 1:100), CD45 (ab8216, 1:50), CD34 (ab8536, 1:50), CD14 (ab182032, 1:200), CD19 (ab31947, 1:50), HLA-DR (ab20181, 1:50), and goat anti-mouse IgG isotope antibody (1:1000, BD Biosciences Pharmingen, San Jose, CA, USA) conjugated with fluorescein isothiocyanate (FITC). The above antibodies were supplied by Abcam Inc. (Cambridge, MA, UK). The samples were analyzed with the FACSVerse instrument (BD Biosciences Pharmingen, San Jose, CA, USA) with FlowJo software (Tree Star Inc., Ashland, OR, USA).

### Isolation and identification of BMMSC-derived exosomes

The BMMSCs at the logarithmic growth phase were collected, and their secreted exosomes were isolated from the supernatant by gradient centrifugation. The protein concentration of exosomes was determined by the bicinchoninic acid (BCA) assay. Expression of specific surface biomarkers of exosomes (CD63, CD81, TSG101, and calnexin) was detected immunohistochemically. Zetasizer Nano ZS (Malvern Panalytical Ltd., Malvern, UK) was used to determine the particle size of exosomes. The exosome suspension solution was fixed with 2% paraformaldehyde, 2.5% glutaraldehyde, and 1% osmic acid for 1.5 h. The fixed exosomes were dehydrated with gradient ethanol, immersed in epoxy resin overnight, and polymerized at 35, 45, and then 60 °C for 24 h. Lastly, the embedded exosomes were cut into ultrathin slices, stained with lead and uranium salts, and observed under a transmission electron microscope.

### Labeling and tracking of BMMSC-derived exosomes

According to the manufacturer’s protocol, BMMSC-derived exosomes were labeled using CM-Dil (Beyotime Biotechnology Inc., Haimen, China), and then cultured at 37 °C for 30 min in the dark. To remove unbound dye, the exosomes were rinsed with phosphate-buffered saline (PBS) and centrifuged at 100,000×*g* at 4 °C for 70 min and then at 800×*g* for 5 min at room temperature. At last, the exosomes were mixed together and incubated at 37 °C for 24 h. The uptake of dye by exosomes was then observed under a fluorescence microscopy (Leica, Weltzlar, Germany), with image analysis using Leica Application Suite Advanced Fluorescence software.

### Co-culture of BMMSC-derived exosomes and NSCLC cells

Exosomes were extracted from transfected BMMSCs, and then co-cultured with NSCLC cells transfected with NC mimic, miR-144 mimic, NC inhibitor, or miR-144 inhibitor for 48 h.

### 5-Ethynyl-2′-deoxyuridine (EdU) assay

Cells were seeded into 24-well plates, with triplicate wells for each group. Subsequently, the cultured cells were added with EdU (C10341-1, Guangzhou RiboBio Co., LTD., Guangzhou, China) to a final concentration of 10 μM, with culture continuing for another 2 h. Next, the cells were fixed, rinsed, and then incubated with PBS containing 0.5% Triton-100. Suspended cells were then stained with Apollo® 567 (Guangzhou RiboBio Co., LTD, Guangzhou, China) for 30 min and with 1× Hoechst 33342 for 30 min. Finally, the number of positive cells was recorded under a fluorescence microscope (model: FM-600, Shanghai Pudan Optical Instrument Co., Ltd., Shanghai, China).

### Colony formation assay

Cells were seeded in 10 mL culture medium (200 cells/culture dish) and cultured for 3 weeks. When visible clones were observed, cells were fixed and stained with 0.1% crystal violet for 10 min. Finally, the number of clones was counted, while the colony formation rate was calculated as follows: colony formation rate = the number of clones/the number of plated cells × 100%.

### Flow cytometry

Cells (1 × 10^6^ cells/mL) were fixed with 70% precooled ethanol at 4 °C overnight. Next, 100 μL portions of cell suspension was stained with 50 μg propidium iodide (PI) dye liquor (40710ES03, Shanghai Qcbio Science & Technologies CO., Ltd., Shanghai, China) containing ribonuclease at 4 °C for 30 min in the dark. Finally, cell cycle was recorded by detecting (red) fluorescence at 488 nm in conjunction with flow cytometry (BD, FL, NJ, USA) after filtering of the cell suspension through 100 mesh nylon.

### Xenograft tumor model

Fifteen BALB/c-nu female nude mice (5–6 weeks, 18–22 g) were randomly allocated to three groups of five animals each. NSCLC cell suspension (1 × 10^7^ cells/mouse) was subcutaneously injected into each mouse to establish the subcutaneous xenograft tumor model. When the mean tumor volume reached 100 mm^3^, the BMMSC-secreted exosomes (100 μL, concentration of 1 μg/μL) were injected into the tail vein of each mouse on the 5th, 10th, 15th, and 20th days. The injections consisted of (1) normal saline (control group), (2) exosomes carrying negative control of miR-144 agomir (Exo-NC-agomir group), and (3) exosomes carrying miR-144 agomir (Exo-miR-144 agomir group). Tumor growth (volume and weight) was observed, and tumor volume (mm^3^) was calculated as 0.5 × the long axis (mm) × the short axis^2^ (mm^2^). Twenty-five days later, all nude mice were euthanized and their tumor tissues were isolated. Histopathological analysis of the tumor tissue was performed after paraffin embedding and slicing.

### Statistical analysis

Statistical analyses were performed using SPSS 21.0 statistical software (IBM Corp., Armonk, NY, USA). Measurement data were presented as mean ± standard deviation. The paired *t* test was applied for comparisons between paired data conforming to the homogeneity of variance and normal distribution, while the unpaired *t* test was used for comparisons between unpaired data conforming to the homogeneity of variance and normal distribution. Data among multiple groups were analyzed by one-way analysis of variance (ANOVA), followed by a Tukey multiple comparisons *post*-test. Data among multiple groups at different time points were measured by repeated measures ANOVA, followed by Bonferroni post hoc test. A value of *p* < 0.05 was statistically significant.

## Results

### CCNE1 and CCNE2 were highly expressed in NSCLC tissues and cell lines

**Fig. 1:**
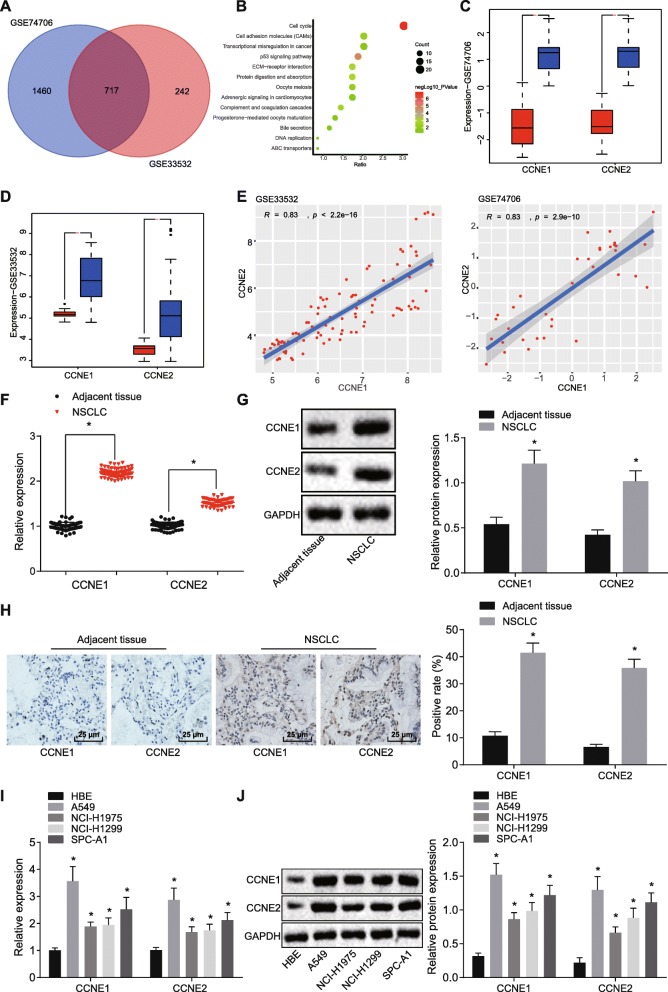
High expression of CCNE1 and CCNE2 in NSCLC tissues and cells. **a** Comparisons of differently expressed genes in the expression profile of NSCLCs GSE74706 and GSE33532. **b** KEGG pathway enrichment of the DEGs in NSCLC analyzed by DAVID software. **c**, **d** Expression of CCNE1 and CCNE2 in expression profile of NSCLC GSE74706 and GSE33532. **e** Correlation analysis of expression of CCNE1 and CCNE2. **f** The expression of CCNE1 and CCNE2 in NSCLC tissues and adjacent normal tissues determined by RT-qPCR. **g** The expression of CCNE1 and CCNE2 protein in NSCLC tissues and adjacent normal tissues tested by western blot analysis. **h** Expression of CCNE1 and CCNE2 in NSCLC tissues detected by IHC (× 400). *p < 0.05 vs. the adjacent normal tissues. **i** The expression of CCNE1 and CCNE2 in different NSCLC cell lines measured by RT-qPCR. **j** The expression of CCNE1 and CCNE2 in different NSCLC cell lines assessed by western blot analysis. *p < 0.05 vs. the HBE cell line. The above measurement data were expressed as mean ± standard deviation. Comparisons between tumor tissues and adjacent normal tissues were analyzed by paired *t* test. Comparisons between two groups were analyzed by unpaired *t* test. The experiment was repeated in triplicate

The difference analysis was performed on expression profiles of NSCLC (GSE74706 and GSE33532), and 717 common DEGs were obtained based on the Venn diagram (Fig. 1a). The DEGs were enriched in signaling pathways involved in cell cycle, the p53 signaling pathway, and ECM-receptor interaction (Fig. 1b). CCNE1 and CCNE2, which belong to the cyclin family, participate in regulating cell cycle, the p53 signaling pathway, and oocyte meiosis [14]. Therefore, we investigated whether CCNE1 and CCNE2 were involved in NSCLC.

Both CCNE1 and CCNE2 proved to be highly expressed in NSCLC GSE74706 (Fig. 1c) and GSE33532 (Fig. 1d). The expressions of CCNE1 and CCNE2 were positively correlated in the expression profile of GSE74706 and GSE33532 (Fig. 1e). Levels of CCNE1 and CCNE2 in NSCLC tissues were higher than those in adjacent normal tissues (*p* < 0.05) (Fig. 1f, g). Meanwhile, IHC demonstrated that, in comparison with adjacent normal tissues, CCNE1 and CCNE2 expressions were elevated and that CCNE1 and CCNE2 were located in the nucleus (*p* < 0.05) (Fig. 1h). Furthermore, expressions of CCNE1 and CCNE2 in NSCLC cell lines (A549, NCI-H1975, NCI-H1299, SPC-A1) were much higher than in normal HBE cells (*p* < 0.05). Among the four NSCLC cell lines examined, A549 cells were found to have the highest expression of CCNE1 and CCNE2 (*p* < 0.05) (Fig. 1i, j).

### Silencing of CCNE1 and CCNE2 inhibited cell proliferation, colony formation, and the number of S phase-arrested cells in NSCLC cells

**Fig. 2:**
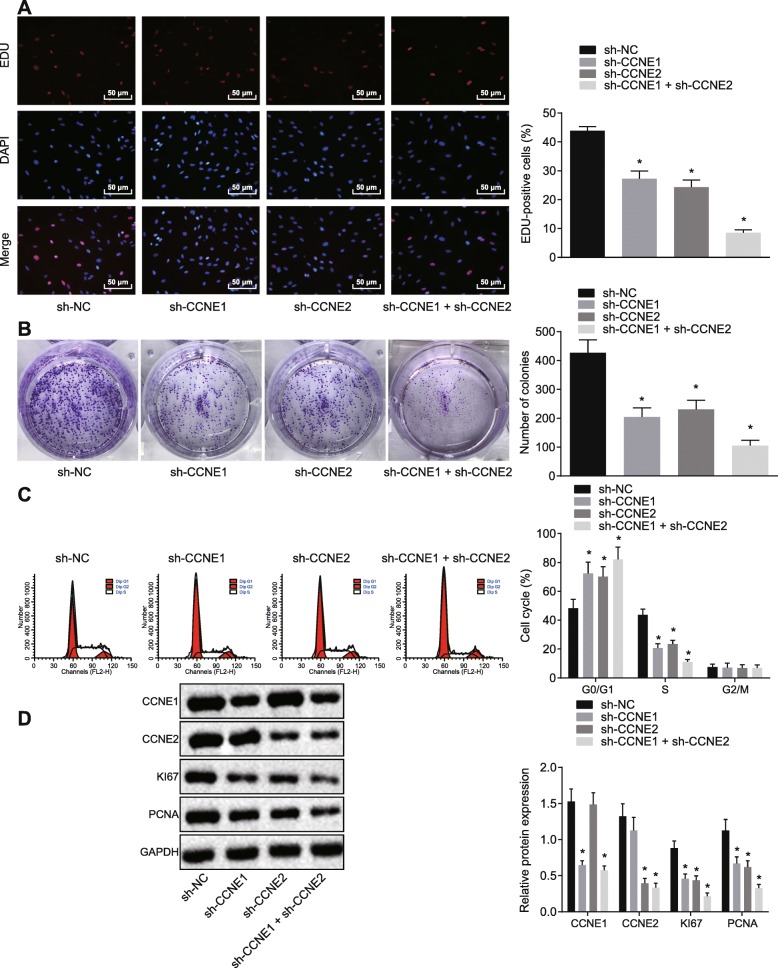
Silencing CCNE1 and CCNE2 inhibited proliferation, colony formation, and reduced the number of S phase-arrested cells cultured NSCLC cells. **a** Cell proliferation measured by EdU assay (× 200). **b** Number of colonies formed measured by colony formation assay. **c** Cell cycle progression detected by flow cytometry. **d** The expression of proliferation related factors assessed by western blot analysis. *p < 0.05 vs. A549 cells transfected with sh-NC. The above measurement data were expressed as mean ± standard deviation. Data among multiple groups were analyzed by one-way ANOVA, followed by a Tukey post hoc test. The experiment was repeated in triplicate

EdU assay and colony formation assay showed that A549 cells transfected with sh-CCNE1, sh-CCNE2, or co-transfected with sh-CCNE1 and sh-CCNE2 exhibited restrained abilities of proliferation and colony formation, with co-silencing CCNE1 and CCNE2 showing the strongest effect (Fig. 2a, b). The flow cytometry assay indicated that inhibiting CCNE1 or CCNE2 arrested cells at the G0/G1 phase, while notably decreasing the number of cells arrested at S phase (*p* < 0.05) (Fig. 2c). Expression of proliferation-related factors (KI67, PCNA) in A549 cells was reduced by sh-CCNE1 and sh-CCNE2, with the lowest expression after co-transfection with both (Fig. 2d).

### MiR-144 is poorly expressed in NSCLC tissues and cell lines and could target CCNE1 and CCNE2

**Fig. 3:**
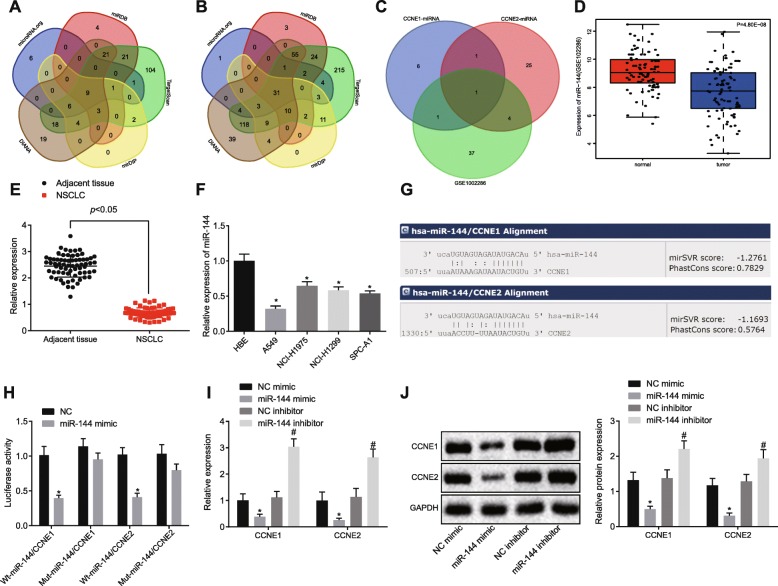
MiR-144 targets and negatively regulates CCNE1 and CCNE2. **a**, **b** Prediction of upstream targeted miRNAs of CCNE1 and CCNE2. **c** Comparison between upstream miRNAs of CCNE1 and CCNE2 and differentially expressed miRNAs predicted from GSE102286. **d** Expression of miR-144 in GSE102286. **e** The expression of miR-144 in NSCLC tissues and adjacent normal tissues tested by RT-qPCR. *p < 0.05 vs. the adjacent normal tissues. **f** The expression of miR-144 in NSCLC cell lines identified by RT-qPCR. *p < 0.05 vs. the HBE cell line. **g** The binding sites of miR-144 on CCNE1 and CCNE2 predicted by the bioinformatics websites (http://www.microrna.org/microrna/home.do). **h** The binding of miR-144 to CCNE1 and CCNE2 confirmed by dual luciferase reporter assay. *p < 0.05 vs. A549 cells transfected with NC. **i** The expression of CCNE1 and CCNE2 assessed by RT-qPCR. **j** The expression of CCNE1 and CCNE2 detected by western blot analysis. *p < 0.05 vs. A549 cells transfected with NC mimic. ^#^*p* < 0.05 vs. A549 cells transfected with NC inhibitor. Comparisons between two groups were analyzed by unpaired *t* test. Data among multiple groups were analyzed by one-way ANOVA, followed by a Tukey post hoc test. The experiment was repeated in triplicate

Furthermore, upstream regulated miRNAs of CCNE1 and CCNE2 were predicted based on the databases microRNA.org, miRDB, TargetScan, mirDIP, and DIANA. The comparison of prediction results in the Venn diagram indicated nine intersecting miRNAs for CCNE1 (Fig. 3a) and 31 for CCNE2 (Fig. 3b). There was only one intersecting miRNA, hsa-miR-144-3p (Fig. 3c), suggesting that miR-144 may play a role in NSCLC by targeting CCNE1 and CCNE2. The low expression of miR-144 in GSE102286 showed that miR-144 was poorly expressed in NSCLC tissues (Fig. 3d).

In addition, RT-qPCR revealed that, compared with the adjacent normal tissues, miR-144 expression in resected NSCLC tissues was downregulated (*p* < 0.05) (Fig. 3e). RT-qPCR also revealed that, compared with normal HBE cells, the expression of miR-144 was reduced in NSCLC cell lines A549, NCI-H1975, NCI-H1299, and SPC-A1, while being most significantly decreased in A549 cells (*p* < 0.05) (Fig. 3f).

The presence of specific binding sites of miR-144 on CCNE1 and CCNE2 was predicted by the bioinformatics database (http://www.microrna.org/microrna/home.do) (Fig. 3g). Then, a dual luciferase reporter assay suggested that, in contrast to transfection of NC, the luciferase activities of WT-miR-144/CCNE1 and WT-miR-144/CCNE2 were lowered (*p* < 0.05) (Fig. 3h). Moreover, compared with the transfection of NC mimic, expressions of CCNE1 and CCNE2 were reduced by miR-144 mimic, but upregulated by miR-144 inhibitor, compared with the transfection of NC inhibitor (*p* < 0.05) (Fig. 3i, j). Thus, miR-144 was poorly expressed in NSCLC, and miR-144 could negatively regulate expressions of CCNE1 and CCNE2.

### Elevated miR-144 levels impeded cell proliferation, colony formation, and S phase-arrested cells in NSCLC cells

**Fig. 4:**
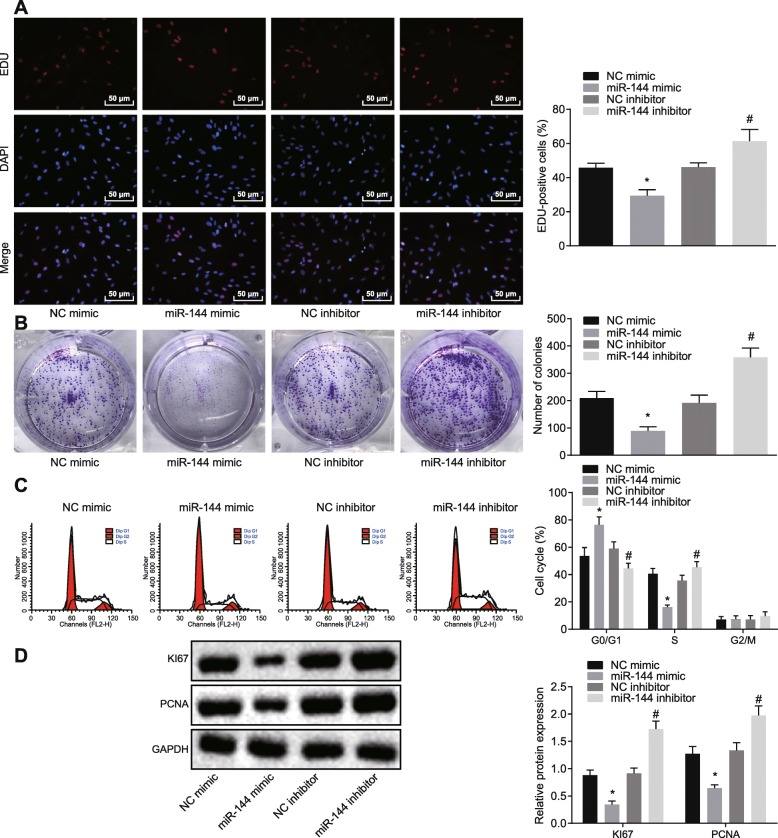
Overexpression of miR-144 attenuates NSCLC cell proliferation and colony formation and reduces the number of S phase-arrested cells. **a** Cell proliferation detected by EdU assay (× 200). **b** Number of colony formation assessed by colony formation assay. **c** Cell cycle progression measured by flow cytometry. **d** The expression of proliferation-related factors determined by western blot analysis. *p < 0.05 vs. A549 cells transfected with NC mimic. ^#^*p* < 0.05 vs. A549 cells transfected with NC inhibitor. The above measurement data were expressed as mean ± standard deviation. Comparisons between two groups were analyzed by unpaired *t* test. The experiment was repeated in triplicate

In contrast to findings with NC mimic, the proliferation and colony formation abilities were lowered by miR-144 mimic (*p* < 0.05) (Fig. 4a, b). The number of G0/G1 phase-arrested cells was increased, and S phase-arrested cells were reduced in response to miR-144 mimic treatment (Fig. 4c). Meanwhile, expression of KI67 and PCNA was notably downregulated in response to miR-144 mimic (*p* < 0.05) (Fig. 4d). In conclusion, overexpression of miR-144 could lead to suppressed cell proliferation and colony formation as well as elevated G0/G1 phase-arrested cells and lowered S phase-arrested cells in NSCLC cells.

### miR-144 suppressed NSCLC cell proliferation, colony formation, and S phase-arrested cells by targeting CCNE1 and CCNE2

**Fig. 5:**
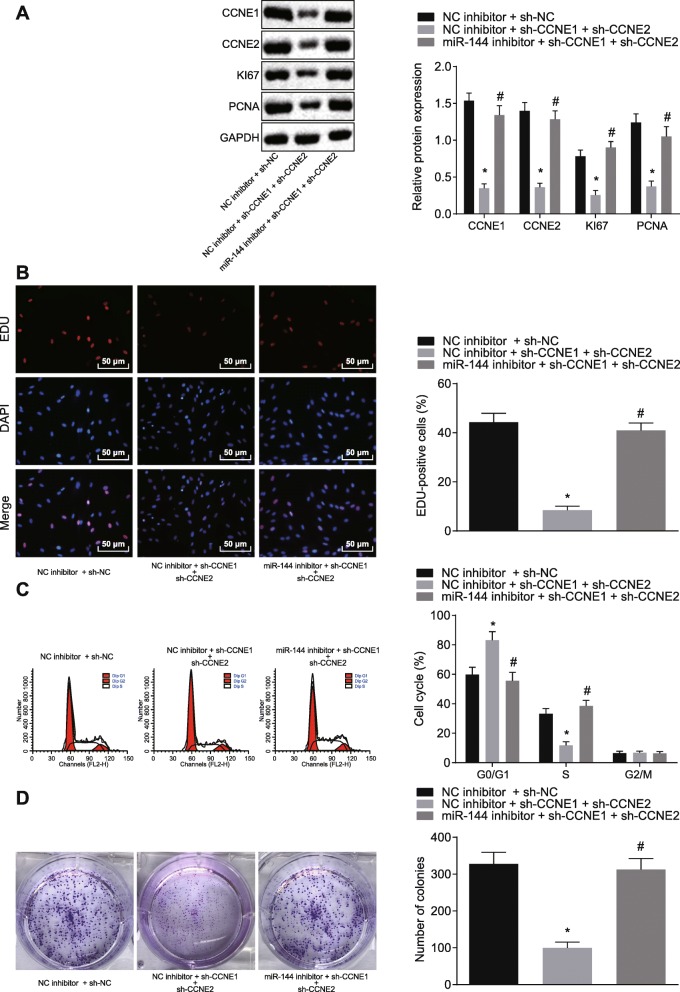
miR-144 impedes NSCLC cell proliferation and colony formation and reduces the number of S phase-arrested cells by downregulating CCNE1 and CCNE2. **a** The expression of proliferation-related factors detected by western blot analysis. **b** Cell proliferation assessed by EdU assay (× 200). **c** Cell cycle progression identified by flow cytometry. **d** Number of colony formation assessed by colony formation assay. *p < 0.05 vs. A549 cells co-transfected with NC inhibitor + sh-NC. ^#^*p* < 0.05 vs. A549 cells co-transfected with NC inhibitor + sh-CCNE1 + sh-CCNE2 group. The above measurement data were expressed as mean ± standard deviation. Data among multiple groups were analyzed by one-way ANOVA, followed by a Tukey post hoc test. The experiment was repeated in triplicate

In order to test whether miR-144 regulated NSCLC progression by targeting CCNE1 and CCNE2, miR-144 was overexpressed in A549 cells with or without co-expressing CCNE1 and CCNE2. Western blot analysis indicated that the expressions of KI67 and PCNA were decreased after overexpressing miR-144 but that this effect was blocked by co-expressing of CCNE1 and CCNE2 (*p* < 0.05) (Fig. 5a).

The EdU and colony formation assays showed that miR-144 mimic could reduce cell proliferation and colony formation ability, an effect that was rescued by co-transfection of CCNE1 and CCNE2 (*p* < 0.05) (Fig. 5b, c). Cell cycle progression detected by flow cytometry revealed that the number of G0/G1 phase-arrested cells was enhanced while the number of S phase-arrested cells was reduced by overexpression of miR-144, an effect which could be reversed by overexpression of CCNE1 and CCNE2 at the same time (*p* < 0.05) (Fig. 5d). Thus, miR-144 could decrease cell proliferation, colony formation, and the number S phase-arrested cells and elevate G0/G1 phase-arrested NSCLC cells by inhibiting CCNE1 and CCNE2.

### Characterization of BMMSC-derived exosomes

**Fig. 6:**
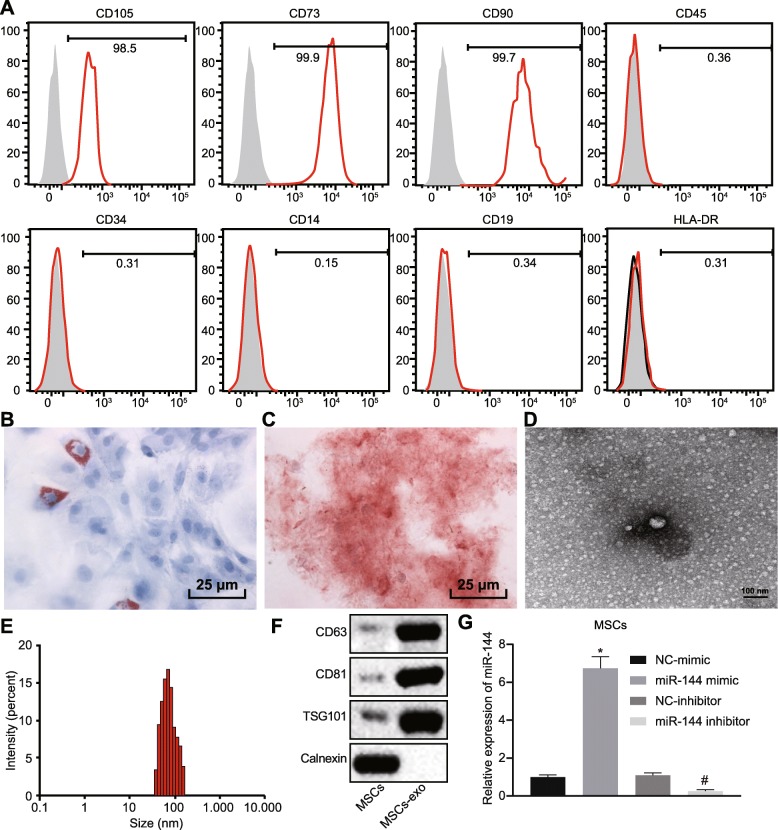
Isolation and identification of BMMSC-derived exosomes. **a** Expression of BMMSC surface markers determined by flow cytometer. **b** Adipogenic differentiation of BMMSCs detected by oil red o staining (scale bar = 25 μm). **c** Osteogenic differentiation of BMMSCs tested by alizarin red staining (scale bar = 25 μm). **d** BMMSC-derived exosomes observed under the transmission electron microscope (scale bar = 100 nm). **e** Particle distribution of BMMSC-derived exosomes analyzed by Zetasizer Nano ZS. **f** Expression of BMMSC surface markers measured by western blot analysis. **g** miR-144 expression in BMMSCs in response to miR-144 mimic/inhibitor treatment as detected by RT-qPCR. The above measurement data were expressed as mean ± standard deviation. Data among multiple groups were analyzed by one-way ANOVA, followed by a Tukey post hoc test. The experiment was repeated in triplicate

BMMSCs were isolated from the bone marrow and subjected to flow cytometry for detection of BMMSC surface markers. As shown in Fig. 6a, the putative BMMSCs isolated from the patient’s bone marrow were positive for CD105, CD73, and CD90 and negative for CD45, CD34, CD14, CD19, and HLA-DR, thus affirming their identity as BMMSCs. The isolated BMMSCs were cultured in specific culture medium for 2 weeks to induce adipogenic differentiation, which was confirmed by the presence in the cells of red liquid droplets to oil red O staining (Fig. 6b). Moreover, osteogenic differentiation of BMMSCs was confirmed by presence of numerous red nodules with unclear cell structure and abundant red calcium deposition after alizarin red staining at 2 weeks after differentiation (Fig. 6c). Thus, the isolated cells were MSCs.

The exosomes isolated from BMMSCs were round and oval membranous vesicular disc structures, with intact capsule and similar morphology (Fig. 6d). Particle size of BMMSC-derived exosomes as analyzed by Zetasizer Nano ZS showed a mean diameter of approximately 62 nm (Fig. 6e). Expression of CD81, CD63, and TSG101 protein in BMMSC-derived exosomes was much higher than that in BMMSCs (Fig. 6f). In order to further explore the effect of BMMSC-derived exosomal miR-144 on NSCLC, we first transfected miR-144 mimic or inhibitor into BMMSCs. miR-144 expression in BMMSCs was determined by RT-qPCR (Fig. 6g), which showed that the miR-144 expression was appreciably elevated in response to miR-144 mimic treatment (versus NC mimic treatment) and decreased in response to miR-144 inhibitor treatment (versus NC inhibitor treatment) (*p* < 0.05). Hence, BMMSC-derived exosomes were isolated successfully.

### BMMSC-derived exosomes restrained cell proliferation and colony formation in NSCLC cells through delivery of miR-144

**Fig. 7:**
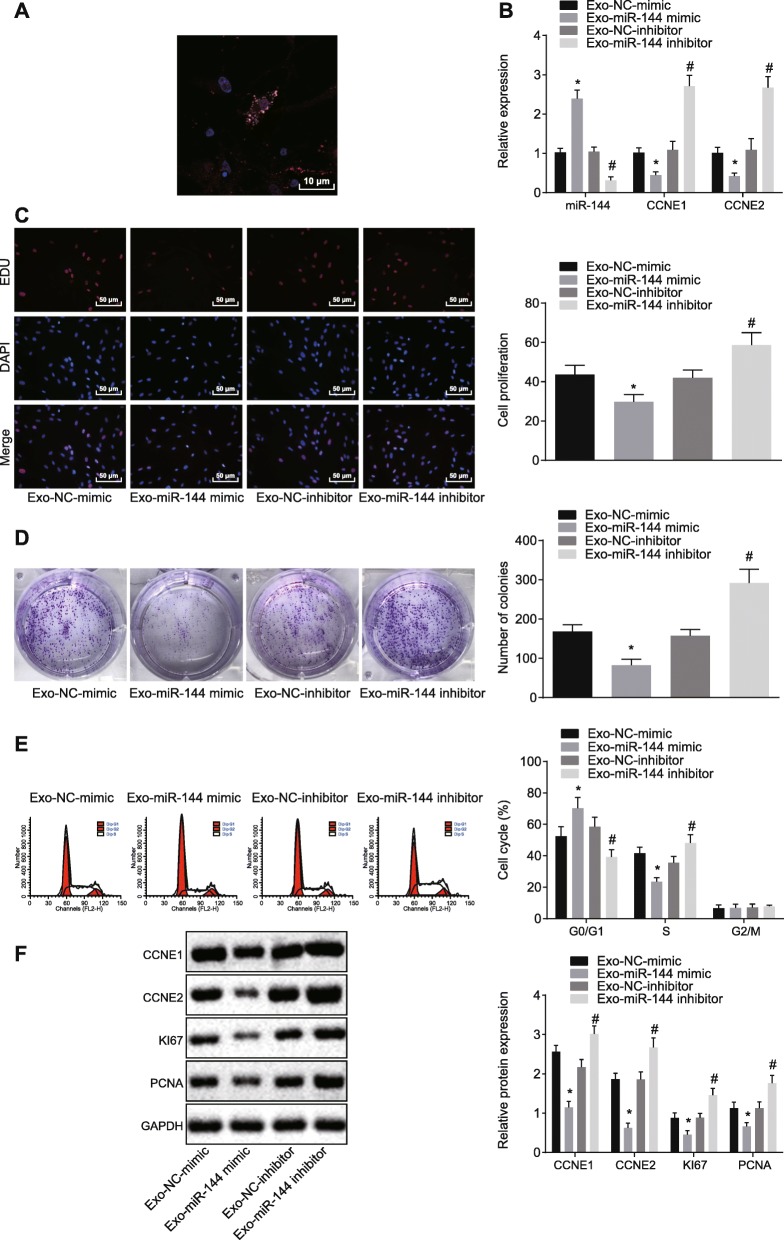
BMMSC-derived exosomes suppress cell proliferation and colony formation through delivering miR-144 to A549 cells. **a** Uptake of exosomes by A549 cells observed under a fluorescence microscope (× 1000). **b** Relative expression of miR-144, CCNE1, and CCNE2 detected by RT-qPCR. **c** Cell proliferation assessed by EdU assay (× 200). **d** Number of colony formation identified by colony formation assay. **e** Cell cycle progression measured by flow cytometry. **f** Relative expression of proliferation related factors tested by western blot analysis. *p < 0.05 vs. A549 cells transfected with Exo-NC mimic. ^#^*p* < 0.05 vs. A549 cells transfected with Exo-NC inhibitor. The above measurement data were expressed as mean ± standard deviation. Comparisons between two groups were analyzed by unpaired *t* test. The experiment was repeated in triplicate

BMMSC-derived exosomes labeled with Dil were co-cultured with A549 cells. The exosome uptake of A549 cells under fluorescence microscopy suggested that after 48 h of co-culture, the exosome uptake of A549 cells was increased (Fig. 7a). RT-qPCR revealed that, compared with co-culture of Exo-NC mimic, miR-144 was highly expressed and CCNE1 and CCNE2 were poorly expressed in A549 cells after co-culture with Exo-miR-144 mimic (*p* < 0.05) (Fig. 7b). In addition, in contrast to Exo-NC mimic results, cell proliferation and colony formation rate of A549 cells were decreased in response to Exo-miR-144 (*p* < 0.05) (Fig. 7c, d). Compared with Exo-NC mimic, the number of G0/G1 phase-arrested cells was increased, S phase-arrested cells were decreased, and expression of CCNE1, CCNE2, KI67, and PCNA was lower in A549 cells co-cultured with Exo-miR-144 (Fig. 7e, f).

### BMMSC-derived exosomes inhibited the growth of xenografted tumors in nude mice through delivery of miR-144 to NSCLC cells

**Fig. 8:**
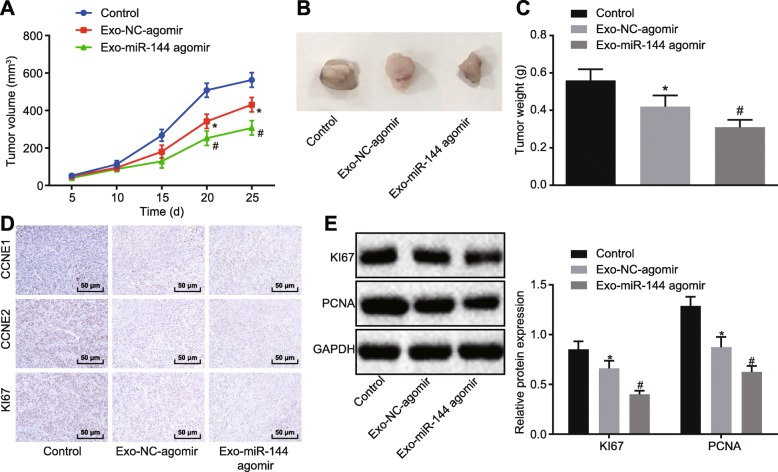
BMMSC-derived exosomes inhibit the growth of xenografted tumors in nude mice by delivering miR-144. **a** Volume growth of xenograft tumors in mice. **b** Representative images of xenograft tumors in mice. **c** Weight of xenograft tumors in mice. **d** Expression of CCNE1, CCNE2, and KI67 in xenograft tumors assessed by IHC (× 200). **e** The expression of KI67 and PCNA in mice determined by western blot analysis. *p < 0.05 vs. nude mice injected with Exo-NC-agomir. The above measurement data were expressed as mean ± standard deviation. Data among multiple groups were analyzed by one-way ANOVA, followed by a Tukey post hoc test. Data among multiple groups at different time points were measured by repeated measures ANOVA, followed by Bonferroni post hoc test. *N* = 5

To prove further the inhibitory effect of BMMSC-derived exosomes on tumor formation of A549 cells in vivo, A549 cells were injected subcutaneously into nude mice to establish the xenograft tumor model, while exosomes were injected through a tail vein. Then, the formation of xenografted tumor was observed and recorded. Compared with Exo-NC-agomir treatment, the tumor volume and weight of xenograft tumors were reduced after injection of Exo-miR-144 agomir (*p* < 0.05) (Fig. 8a–c). IHC revealed that positive rates of CCNE1, CCNE2, and KI67 were reduced in the xenografted tumor tissues upon the injection of Exo-miR-144 agomir compared with the injection of Exo-NC-agomir (*p* < 0.05) (Fig. 8d). Next, western blot analysis showed that expression of KI67 and PCNA in tumor tissues was downregulated in response to injection with Exo-miR-144 agomir, compared to findings with Eco-NC agomir (*p* < 0.05) (Fig. 8e).

## Discussion

NSCLC has various pathological features [15]. NSCLC has a special high tumor mutation burden, which is defined as the number of non-synonymous coding mutations per megabase, especially in smokers [16]. MSC-derived exosomes have been shown to play essential roles in tumorigenesis, angiogenesis, and metastasis and can also present with a tumor-suppressing effect [17]. In the current study, we investigated the effect on NSCLC development of miR-144 shuttled from the BMMSC exosomes, finding that delivery of miR-144 via BMMSC-derived exosomes could downregulate CCNE1 and CCNE2 expression, thus attenuating NSCLC cell proliferation, colony formation, and cell cycle progression.

Our initial findings showed that CCNE1 and CCNE2 were highly expressed while miR-144 was poorly expressed in NSCLC tissues and cell lines. CCNE1 was reported to be repeatedly amplified and/or upregulated in high-grade serous ovarian cancer [18]. The previous literature has confirmed that upregulation of CCNE1 occurs in human hepatocellular carcinomas [19]. High expression of CCNE2 in NSCLC tissues was also demonstrated in a previous report [6]. In addition, decreased miR-144 expression has been found in colorectal cancer tissues [20] and likewise in osteosarcoma cell lines and tissues [21]. In agreement with our findings, low expression miR-144 was revealed in lung adenocarcinoma tissues [22]. Moreover, CCNE1 and CCNE2 were targets of miR-144, which could be inversely regulated by miR-144. Similarly, CCNE1 and CCNE2 have been previously identified as direct targets of miR-144-5p; bladder cancer patients with high CCNE1 or CCNE2 expression have lower overall survival rate than patients with low expression [23], which suggested that downregulation of CCNE1 and CCNE2 inhibited bladder cancer cell proliferation. Consistent with our present results, downregulation of CCNE1 and CCNE2 could inhibit NSCLC cell proliferation, colony formation, and the number of S phase-arrested cells. Also, we find that miR-144 prevented cell proliferation and colony formation as well as S phase-arrest of NSCLC cells by downregulating CCNE1 and CCNE2. A previous literature proved that miR-144 delivery restrained growth and induced apoptosis of NSCLC cells [24]. Ki67 is a proliferation marker [25] and PCNA is protein needed for cell cycle progression from G1 to S phase [26]. Our study also revealed that overexpression of miR-144 could decrease the expression of the markers Ki67 and PCNA. Taken together, the aforementioned findings suggest an inhibitory role of miR-144 in NSCLC cell biological functions, which is mediated via suppression of CCNE1 and CCNE2.

MSCs are a population of adult stem cells with autophagic and multilineage differentiation capabilities, including to chondrocytes, osteocytes, and adipocytes. The BMMSC secretome can inhibit the growth and promote apoptosis of NSCLC cells [27], and BMMSCs can be identified by their expression of certain surface markers. High expression of CD90 and CD105 and absent expression of CD34 were reported in MSCs in a previous study [28]. Consistently, we have identified that CD105 (76.99 ± 31.05%), CD73 (96.43 ± 3.88%), and CD90 (89.87 ± 8.80%) were positively expressed, while CD45, CD34, and HLA-DR were negatively expressed in BMMSCs [29]. BMMSCs migrate to lung tumors, differentiate into lymphatic endothelial cells, and then participate in lymphangiogenesis [30]. In addition, our earlier work found that BMMSCs can transfer miR-144 to NSCLC cells through transport in exosomes. Indeed, exosomes are not only important in intercellular communication, but can be also used as carriers of therapeutic genes and drugs [31]. miRNAs loaded into exosomes can be delivered to the recipient’s niche cells, where they have a great impact on gene expression regulation [32]. A highly diverse population of miRNAs is observed in exosomes from human adipose mesenchymal stem cells, whose derived exosomal miRNAs are reportedly a critical factor for triggering anti-proliferation signaling to ovarian cancer cells [33]. Moreover, exosomes containing certain miRNAs can contribute to the delayed initiation and progression of chronic obstructive pulmonary disease (COPD) and are consequently widely used as diagnostic and prognostic biomarkers in COPD patients [34]. Thus, in keeping with a broad literature, we now report that BMMSC-derived exosomal miR-144 can impede NSCLC cell proliferation, colony formation, and ectopic tumor growth in nude mice.

## Conclusions

**Fig. 9:**
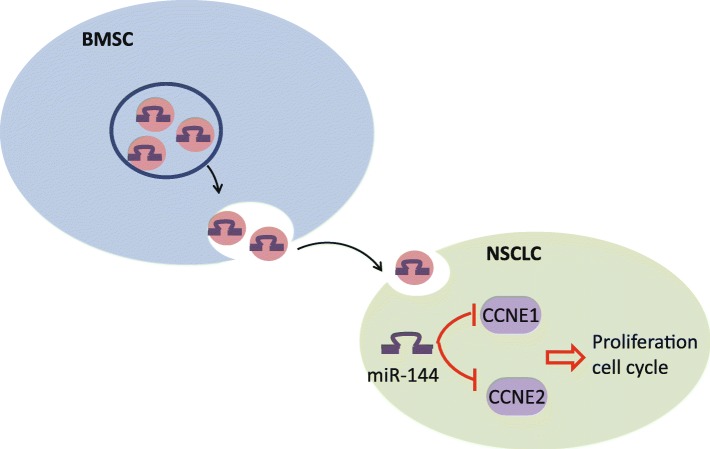
Regulatory mechanism of BMMSC-derived exosomal miR-144 involved in NSCLC cell proliferation and cell cycle progression. BMMSC-derived exosomal miR-144 impeded the proliferation and cell cycle of NSCLC cells by targeting CCNE1 and CCNE2

BMMSC-derived exosomes carrying miR-144 can lower the expression of CCNE1 and CCNE2, thereby inhibiting progression of NSCLC (Fig. 9). The current study sheds new light on the potential of exosomes carrying miR-144 in impacting the development of NSCLC and on the use of miR-144 as new diagnostic and therapeutic biomarkers in the treatment of NSCLC.

## Data Availability

The datasets generated/analyzed during the current study are available.

## Abbreviations

ANOVA: Analysis of variance
BMMSCs: Bone marrow-derived MSCs
CCNE1: Cyclin E1
DEGs: Differentially expressed genes
EdU: 5-ethynyl-2′-deoxyuridine
FBS: Fetal bovine serum
FITC: Fluorescein isothiocyanate
GAPDH: Glyceraldehyde-3-phosphate dehydrogenase
HBE: Human bronchial epithelial
IgG: Immunoglobulin G
IHC: Immunohistochemistry
MEM: Minimum essential medium
miRs or miRNAs: microRNAs
MSCs: Mesenchymal stem cells
MUT: Mutant
NC: Negative control
NSCLC: Non-small cell lung cancer
PBS: Phosphate-buffered saline
PCNA: Proliferating cell nuclear antigen
PI: Propidium iodide
RT-qPCR: Reverse transcription quantitative polymerase chain reaction
sh: Short hairpin
UTR: Untranslated region
WT: Wild type
ZYG11A: ZYG-11 family member A
